# Can the apparent transverse relaxation rate (R2^*^) evaluate the efficacy of concurrent chemoradiotherapy in locally advanced nasopharyngeal carcinoma? a preliminary experience

**DOI:** 10.1186/s12880-023-01029-y

**Published:** 2023-06-01

**Authors:** Xinhua Xu, Ming Chen, Jin Zhang, Yunzhu Jiang, Hua Chao, Jianfeng Zha

**Affiliations:** Department of Radiology, Changzhou Cancer Hospital of Soochow University, 68 Honghe Road, Changzhou, 213000 Jiangsu PR China

**Keywords:** Locoregionally advanced nasopharyngeal carcinoma, Concurrent chemoradiotherapy, Hypoxia, Apparent transverse relaxation rate, Magnetic resonance imaging

## Abstract

**Background:**

The use of the apparent transverse relaxation rate (R2^*^) in nasopharyngeal carcinoma (NPC) has not been previously reported in the literature. The aim of this study was to investigate the role of the R2^*^ value in evaluating response to concurrent chemoradiotherapy (CCRT) in patients with NPC.

**Methods:**

Forty-one patients with locoregionally advanced NPC confirmed by pathology were examined by blood oxygenation level-dependent (BOLD) magnetic resonance imaging (MRI) before and after CCRT, and conventional MRI was performed 3 months after the completion of CCRT. All patients were divided into a responding group (RG) and a nonresponding group (NRG), according to MRI findings 3 months after the end of treatment. The R2^*^ values before (R2*_preT_) and after (R2^*^_postT_) CCRT and the ΔR2^*^ (ΔR2^*^=R2^*^_postT_ – R2^*^_preT_) were calculated in the tumor.

**Results:**

Among the 41 patients, 26 were in the RG and 15 were in the NRG. There was no statistical difference in the R2^*^_preT_ between RG and NRG (*P* = 0.307); however, there were significant differences in R2^*^_postT_ and ΔR2^*^ (*P* < 0.001). The area under the curve of R2^*^_postT_ and ΔR2^*^ for predicting the therapeutic response of NPC was 0.897 and 0.954, respectively, with cutoff values of 40.95 and 5.50 Hz, respectively.

**Conclusion:**

The R2^*^ value can be used as a potential imaging indicator to evaluate the therapeutic response of locoregionally advanced NPC.

## Background

Over 60% of patients with nasopharyngeal carcinoma (NPC) exhibit a locoregionally advanced stage at first diagnosis [1]. Radiotherapy has become a vital treatment approach for locoregionally advanced NPC [2]; however, the 5-year survival rate of patients with locoregionally advanced NPC after radiotherapy is only 50–70% [3]. The main mechanism of function of radiotherapy is that the reactive oxygen species generated by radiation destroy cellular structures [4]. During tumor progression, the abnormal structure and function of blood vessels and tumor cell proliferation lead to an insufficient oxygen supply accompanied by changes in the cancer cell microenvironment, which makes tumors more aggressive [5]. Hypoxia facilitates resistance of tumors against radiation and drug-induced cell death [6], promotes cancer cell proliferation [7], and increases the possibility of recurrence, locoregional spread, and distant metastasis [6, 8]. Therefore, hypoxia-induced treatment resistance is a major cause of treatment failure [9], and it has become an important obstacle to NPC treatment [10, 11]. Establishing a noninvasive and dynamic monitoring method to predict and evaluate the efficacy of NPC treatment is crucial for precision treatment.

Dynamic contrast-enhanced magnetic resonance imaging (DCE-MRI) and diffusion-weighted imaging (DWI) are the commonly used functional MRI techniques to evaluate NPC [12–14]. DCE-MRI provides functional information on neovascularization, and DWI reflects the diffusion characteristics of water molecules. However, they have the disadvantage of being unable to assess changes in oxygenation within the tumor. Blood oxygenation level-dependent MRI (BOLD-MRI), a noninvasive functional MRI to detect the oxygenation level of hemoglobin, indirectly reflects the oxygenation status of tissues [15]. BOLD-MRI uses the difference in tissue susceptibility properties between oxyhemoglobin (diamagnetic) and deoxyhemoglobin (paramagnetic) to express the signal by employing a gradient echo sequence with multiple echo times (TEs) [16]. The apparent transverse relaxation rate (R2^*^) is inversely correlated with the oxygenation level of the tissue [17], and has an approximately linear relationship with the partial pressure of oxygen [18, 19]. R2^*^ values increase when tissue oxygenation levels decrease, and decrease when oxygenation levels increase [20]. Therefore, the R2^*^ value can be used as an index to assess tissue hypoxia; specifically, the higher the R2^*^ signal intensity, the more apparent the tissue hypoxia [21].

Recently, BOLD-MRI technology has become a research hotspot [22–24]. The feasibility of R2^*^ values for evaluating tumor oxygenation status has been demonstrated in studies of breast [25, 26], cervical [22], prostate [19], and rectal cancers [27]. However, there are only few reports on the application of R2^*^ in NPC to date. Since hypoxia is one of the independent risk factors for NPC and R2^*^ values can reflect the oxygenation status near tumor blood vessels, we hypothesized that R2^*^ values would be associated with the final tumor response in patients with NPC receiving concurrent chemoradiotherapy (CCRT). Therefore, this study aimed to investigate the feasibility of the R2^*^ value in evaluating the efficacy of CCRT in patients with locoregionally advanced NPC.

## Materials and methods

### Patients

This study was approved by the ethics committee of our hospital (file number 2022 - SY − 017), and all patients provided informed consent. Forty-one patients with biopsy-proven locoregionally advanced NPC between January 2020 and December 2021 were enrolled in this study. These patients received CCRT, and underwent MRI before and after CCRT.

The inclusion criteria were as follows: (i) biopsy-confirmed NPC that met the criteria of locoregionally advanced NPC at T1-4N2-3M0 according to the 8th edition of the American Joint Committee on Cancer (AJCC) staging criteria for head and neck tumors; and (ii) absence of invasive examination or anticancer treatment history.

The exclusion criteria were as follows: (i) previous chemotherapy and/or radiotherapy history; (ii) interruption of CCRT; (iii) contraindications to MRI examination; and (iv) images that did not meet the requirements of delineating the region of interest (ROI).

All patients received concurrent intensity-modulated radiotherapy and chemotherapy. According to the MRI findings, gross tumor volume (GTVnx) and cervical metastatic lymph node volume (GTVnd) were delineated. Clinical target volume 1 (CTV1) was GTVnx extended by 5–10 mm. Clinical target volume 2 (CTV2) included the potential invasion area of the tumors and the neck lymphatic drainage area requiring prophylactic irradiation. The planning target volume (PTV) was expanded by 3–5 mm in a three-dimensional direction based on the CTV. The prescribed doses to the PTV for GTVnx, GTVnd, CTV1, and CTV2 were 68–70 Gy, 64–68 Gy, 60 Gy, and 54 Gy, respectively. Five sessions per week for a total of 30 sessions were conducted, and all patients received DDP (80 mg/m^2^) chemotherapy. A course of treatment was received every 3 weeks, and a total of 2–3 courses were required.

### MRI protocol

All patients underwent examination on a 3.0 T MR scanner (UMR 780, United-Imaging Healthcare, Shanghai, China) with a 24-channel phased-array head-neck coil. Conventional unenhanced and enhanced MRI scans were performed before and after CCRT, as well as 3 months after CCRT completion. BOLD-MRI was also performed before and after CCRT. The MRI scan parameters were as follows: (i) conventional MRI unenhanced scan with fast spin-echo sequence: axial T1-weighted image (T1WI): repetition time (TR) = 550 ms, echo time (TE) = 13 ms, slice thickness = 5 mm, slice gap = 1 mm, field of view (FOV) = 230 mm × 256 mm, matrix = 320 × 224, flip of angle = 111^°^. Unenhanced axial T2-weighted image: TR = 3014 ms, TE = 79 ms, slice thickness = 5 mm, slice gap = 1 mm, FOV = 230 mm × 230 mm, matrix = 384 × 288, flip of angle = 150^°^. Enhanced T1WI was performed using fat suppression and the remaining parameters were the same as those used in the unenhanced scan. The contrast agent (Gadopentetate dimeglumine injection, Guangdong Kangchen Pharmaceutical Co., LTD., China) at a 0.1 mmol/kg dose was injected intravenously through the median cubital vein with a high-pressure syringe at 2.0 mL/s, followed by 20mL 0.9% normal saline at the same flow rate. (ii) BOLD-MRI: a five echo multi-echo gradient echo sequence: axial: TR = 1177.8 ms, TE = 6.71/13.42/20.13/26.84/33.55 ms, slice thickness = 5 mm, slice gap = 1 mm, FOV = 360 mm × 270 mm, matrix = 256 × 218, flip of angle = 60^°^.

### Image analysis and data measurement

After data acquisition, the BOLD-MRI images were transferred to a post-processing workstation. Data analysis was performed using software (uWS-MR, version R003; United-Imaging Medical) provided by the manufacturer. The software automatically generated color-coded maps of R2^*^; the colors from blue to red represented the R2^*^ values from low to high on R2^*^ maps. Axial sections of the tumor on enhanced T1WI and R2^*^ maps were chosen for imaging analysis. ROI was manually delineated along the margin of the mass on the largest section. The R2^*^ values of the tumor before and within one week after the end of treatment (R2^*^_preT_ and R2^*^_postT_, respectively), and the ΔR2^*^ (ΔR2^*^ = R2^*^_postT_ – R2^*^_preT_), were used for analysis. To test the interobserver reproducibility, ROIs were drawn independently by two radiologists (observers 1 and 2) with 10 and 5 years of experience, respectively. If there was a good level of agreement, the value of observer 1 was used for statistical analysis.

### Response evaluation

Tumor size was defined as the largest diameter of mass measured on axial- enhanced T1WI. The results of the MRI examination 3 months after the end of treatment show that the tumors were divided into a responding group (RG) and a nonresponding group (NRG), as assessed by the Response Evaluation Criteria in Solid Tumors (version 1.1) as follows: RG: complete response or partial response after treatment; NRG: progressive disease or stable disease after treatment.

### Statistical analysis

The IBM SPSS software (version 26.0; SPSS Inc., Chicago, IL, USA) was used to conduct statistical analysis. Normally distributed data were represented as mean ± standard deviation, whereas non-normally distributed data were represented as median (interquartile range). The intraclass correlation coefficient (ICC) was used to compare the inter-observer agreement. The agreement was scored as excellent (ICC > 0.75), moderate (ICC = 0.50–0.75), or poor (ICC < 0.50). The chi-square test was used to analyze sex and AJCC-TNM stage. R2^*^_preT_, R2^*^_postT_, and ΔR2^*^ were compared between RG and NRG using independent samples t-test or Mann–Whitney U test depending on the normality of data distribution. The receiver operating characteristic curve (ROC) was used to analyze and calculate the area under the ROC curve (AUC), sensitivity, and specificity, using the MedCalc 15.6.1 software. The cutoffs of RG and NRG were obtained by calculating the Youden index (Youden index = sensitivity + specificity — 1). The Delong test was used to compare the differences in AUC between the two groups. *p* < 0.05 was considered statistically significant.

## Results

### Clinical features

Overall, 41 patients (24 male and 17 female) were enrolled in the study, with a mean age of 52.20 ± 12.48 years (26–78 years). Tumors were evaluated 3 months after the completion of CCRT, and 26 and 15 patients were in RG and NRG, respectively. Table 1 shows the age, sex, and AJCC-TNM (tumor, node, metastasis) stage characteristics of all patients.

**Table 1:** Clinical data and staging of patients in RG and NRG

	RG (n = 26)	NRG (n = 15)	t/x^2^	*p*
Age	51.88 ± 11.74	52.73 ± 14.08	–0.207^#^	0.837
Sex			3.349^Δ^	0.067
Male	18	6		
Female	8	9		
AJCC-TNM stage			0.241^Δ^	0.887
I	5	2		
II	11	7		
IVa	10	6		

Data are reported as mean ± SD

^#^Independent-samples t-test; ^Δ^Chi-squared test

RG, responding group; NRG, nonresponding group; AJCC, American Joint Committee on Cancer

### Comparison of interobserver agreement

The inter-observer agreement of R2^*^ values measured by observer 1 and observer 2 was excellent (ICC = 0.801, *p* < 0.0001).

### Comparison of parameters between RG and NRG

**Fig. 1:**
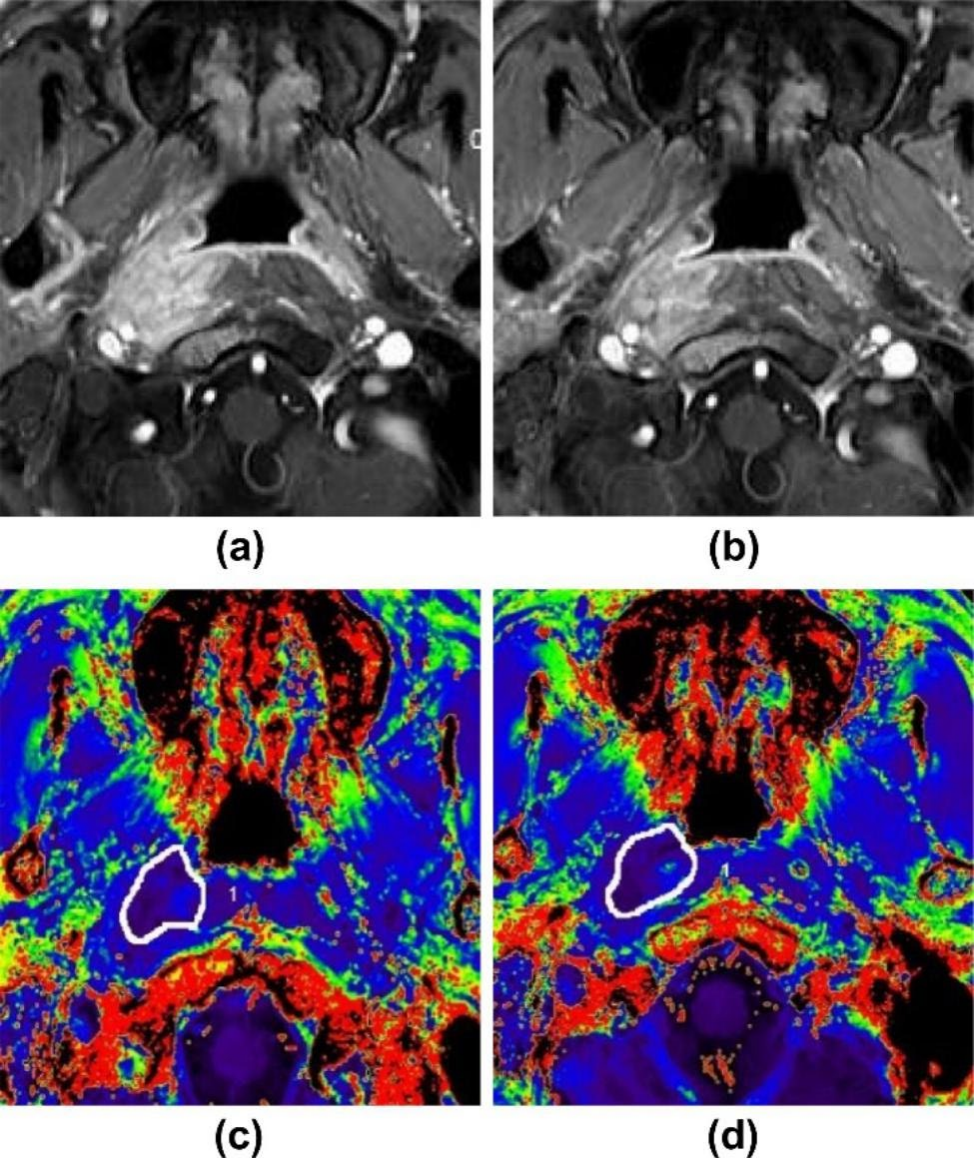
A young man with nasopharyngeal carcinoma (AJCC stage III). **(a)** Axial enhanced fat suppression T1WI before CCRT with lesions located on the right side. **(b)** Axial enhanced fat suppression T1WI at 3 months after the end of CCRT indicates stable disease (SD) according to RECIST 1.1; the patient was included in NRG. **(c)** R2^*^ value in the ROI was 36.40 Hz on the R2^*^ color-coded map before CCRT. **(d)** R2^*^ value in the ROI was 34.70 Hz at 3 months after the end of CCRT. CCRT, concurrent chemoradiotherapy; RECIST, response evaluation criteria for solid tumors; NRG, nonresponding group; ROI, region of interest’ AJCC, American Joint Committee on Cancer; T1WI, T1-weighted image

**Fig. 2:**
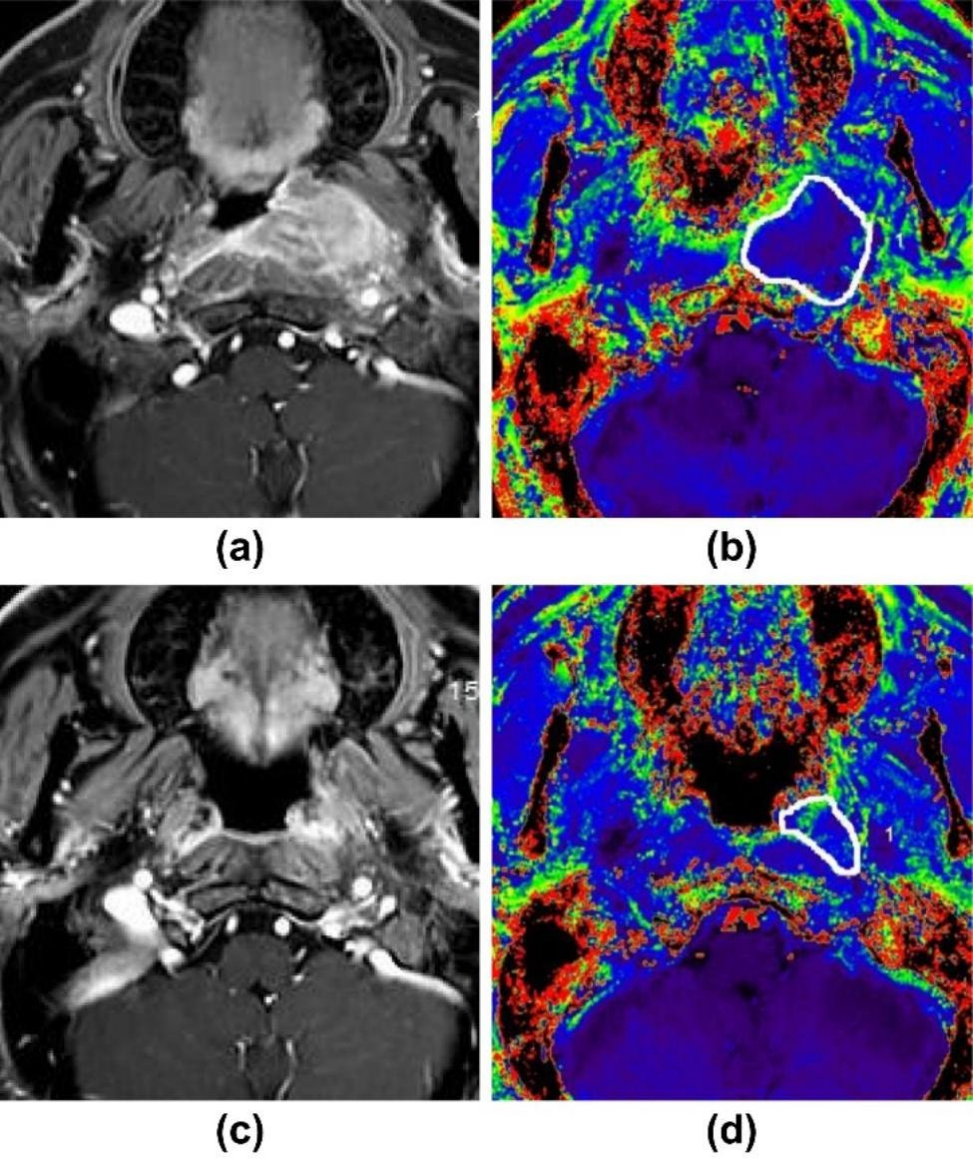
An elderly man with nasopharyngeal carcinoma (AJCC stage III). **(a)** Axial enhanced fat suppression T1WI before CCRT with lesions located on the left side. **(b)** Axial enhanced fat suppression T1WI at 3 months after the end of CCRT indicates a partial response (PR) according to RECIST 1.1; the patient was included in RG. **(c)** R2^*^ value in the ROI was 45.90 Hz on the R2^*^ color-coded map before CCRT. **(d)** R2^*^ value in the ROI was 66.40 Hz at 3 months after the end of CCRT. CCRT, concurrent chemoradiotherapy; RECIST, response evaluation criteria for solid tumors; RG, responding group; ROI, region of interest; AJCC, American Joint Committee on Cancer; T1WI, T1-weighted image

Table 2 shows that R2^*^_preT_ was not significantly different between RG and NRG; however, R2^*^_postT_ and ΔR2^*^ were significantly different. After CCRT, the average R2^*^ value decreased in NRG (Fig. 1) and increased in RG (Fig. 2).

**Table 2:** Comparison of parameters between RG and NRG

	RG (n = 26)	NRG (n = 15)	*t/U*	*p*
R2^*^_preT_ (Hz)	43.78 ± 12.00	48.02 ± 13.69	–1.034^Δ^	0.307
R2^*^_postT_ (Hz)	58.23 ± 12.13	37.83 ± 9.62	5.595^Δ^	< 0.001
ΔR2^*^ (Hz)	16.10 (20.6–12.43)	–10.19 ± 7.57	372^#^	< 0.001

^Δ^Independent-samples t-test (data are reported as mean ± SD); ^#^Mann–Whitney U test (data are reported as median (3rd quartile–1st quartile)

RG, responding group; NRG, nonresponding group; R2^*^_preT_, R2^*^ value of tumor before CCRT; R2^*^_postT_, R2^*^ value of tumor after CCRT; ΔR2^*^, R2^*^_postT_ – R2^*^_preT_; CCRT, concurrent chemoradiotherapy

### Diagnostic performance of R2^*^ values

**Fig. 3:**
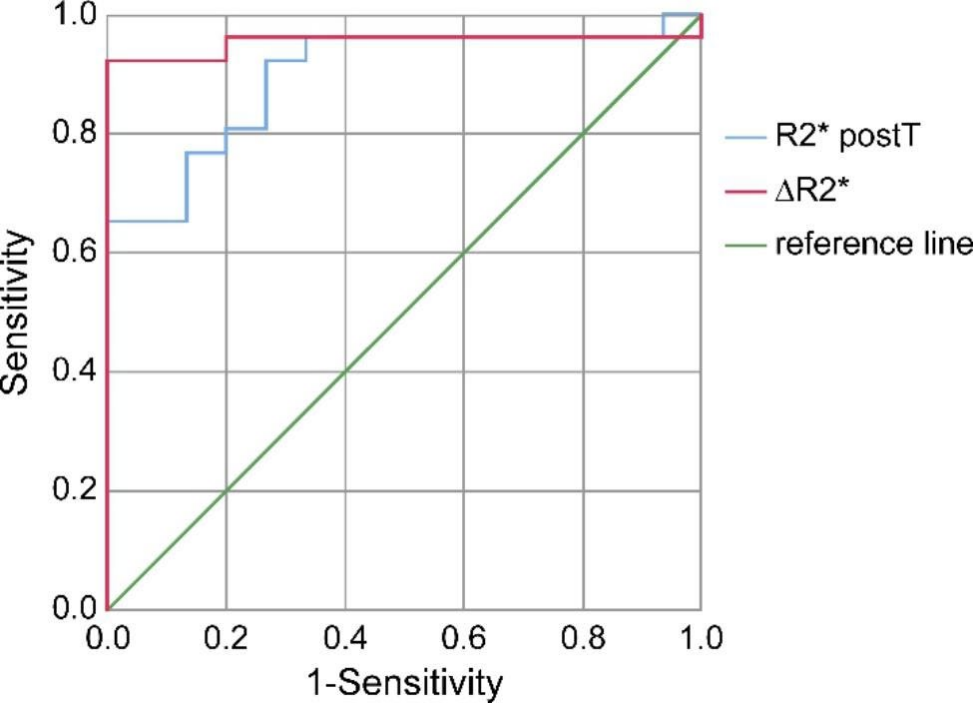
The area under the ROC curve on R2^*^_postT_ and ΔR2^*^ for differentiating the therapeutic response to CCRT. ROC, receiver operating characteristic; CCRT, concurrent chemoradiotherapy

The AUC of R2^*^_postT_ and ΔR2^*^ for predicting the therapeutic response of NPC were 0.897 and 0.954, respectively (Fig. 3), and the cutoffs were 40.95 Hz and 5.50 Hz, respectively (Table 3). There was no significant difference in AUC between R2^*^_postT_ and ΔR2^*^ (Z = 1.020, *p* = 0.308).

**Table 3:** Diagnostic efficacy of R2^*^_postT_ and ΔR2^*^ for predicting therapeutic response to CCRT

	AUC	95%CI	Cutoff	Sensitivity (%)	Specificity (%)
R2^*^_postT_ (Hz)	0.897	0.800–0.995	40.95	92.3	73.3
ΔR2^*^ (Hz)	0.954	0.878–1.000	5.50	92.3	100.00

AUC, area under the curve; 95% CI, 95% confidence interval. CCRT, concurrent chemoradiotherapy

## Discussion

The R2^*^ value can reflect the tissue oxygenation level. When the tissue oxygenation level is reduced the deoxyhemoglobin content in the tissue increases, leading to rapid dephasing of regional spins, a decrease in T2^*^ relaxation time, and an increase in R2^*^ (R2^*^ = 1 / T2^*^ relaxation time). Additionally, when the oxygenation level increases, the increase of oxyhemoglobin in the tissue has little or no effect on the homogeneity of the local magnetic field; therefore, the T2^*^ value increases and the R2^*^ value decreases [20]. Presently, research on the R2^*^ value mainly focuses on body tumors, and the related research on head and neck cancer, especially NPC, is rare. Our preliminary results showed that the R2^*^ value could reflect the oxygenation changes before and after CCRT, and the post-treatment R2^*^ and ΔR2^*^ values could predict CCRT response in NPC. Thus, the R2^*^ value may be a useful marker for evaluating and predicting CCRT efficacy in NPC, which can better guide doctors and help them choose the appropriate treatment.

Our study showed that baseline R2^*^ values before NPC treatment did not predict treatment response. Presently, studies on tumor baseline R2^*^ mainly focus on histological grade [28, 29], and there are no reports on the relationship between baseline R2^*^ values and CCRT treatment response in patients with NPC. Kim et al. [22] suggested that there was no significant correlation between the R2^*^ value before treatment and the volume shrinkage rate after treatment in cervical cancer. All cases in our study were squamous cell carcinoma, and the same pathological type may be one of the reasons why the baseline R2^*^ value of NPC cannot predict the therapeutic response.

Our study showed that the R2^*^ value after CCRT and the ΔR2^*^ value were significantly different. This result indicated that although the tumor response to treatment is not correlated with the baseline R2^*^ value, it is correlated with the corresponding R2^*^ value after treatment. The changes in R2^*^ values before and after CCRT indirectly reflected the oxygenation status within the tumor. Studies have shown that hypoxia is an independent factor for treatment resistance and poor prognosis [30, 31]. We observed that the tumor R2^*^ value after treatment increased in the RG whereas it decreased in the NRG, indicating that effective treatment aggravated tumor hypoxia. Galban et al. [32] suggested that local blood perfusion is rapidly reduced and tumor hypoxia is more severe during treatment, especially in the early stage. Studies have shown that R2^*^ values are affected by multiple factors including oxyhemoglobin level, blood volume, and vascular distribution [28]. Increased R2^*^ values are most commonly due to an increase in the paramagnetic iron content of deoxyhemoglobin, and reduced R2^*^ values are often attributed to calcification or edema [33]. We speculate that effective radiotherapy and chemotherapy destroy the endothelial cells of tumor vessels, increase vascular permeability, and allow soluble oxygen in oxygenated hemoglobin to diffuse between the perfused blood vessels and adjacent tissues, thereby resulting in an increase in the content of paramagnetic deoxyhemoglobin in the tumor blood vessels. Concurrently, ferroptosis of hypoxic tumor cells caused by radiotherapy also increases blood iron content, leading to the increase of the R2^*^ value. There was little change in oxyhemoglobin and deoxyhemoglobin levels in treatment-insensitive tumor tissue; thus, there was little change in the homogeneity of the local magnetic field [20]. The decrease in R2^*^ value may be related to tissue edema caused by radiotherapy. Our study showed that the AUC of R2^*^_postT_ and ΔR2^*^ for predicting therapeutic response were 0.897 and 0.954, respectively. The cutoffs of R2^*^_postT_ and ΔR2^*^ for predicting response were 40.95 Hz and 5.50 Hz, respectively, with a sensitivity of 92.3% and specificity of 73.3% and 100%, respectively. This finding suggests that R2^*^ values can be measured immediately after CCRT in NPC cases to predict therapeutic response. Hence, treatment strategies can be adjusted early based on the R2^*^ value, whereas conventional MRI evaluation typically requires 3 months after the end of treatment.

Our study has some limitations that should be acknowledged. First, this was a preliminary study, and the evaluated technique was not compared with other MRI techniques. In the future, it is necessary to combine DCE-MRI, DW-MRI, and other techniques to verify the true role of the R2^*^ value as a potential biomarker in evaluating treatment efficacy in NPC. Second, there is a lack of pathological evidence due to the unresectable nature of NPC. Since the changes in tumor microstructure precede the changes in imaging, it cannot be ignored that some patients with effective treatment may be misclassified as NRG. Finally, small lesions on the nasopharyngeal surface may affect the accuracy of R2^*^ measurements because of artifacts caused by air-tissue interfaces.

## Conclusions

This study demonstrated that the R2^*^ value at the end of CCRT may predict the treatment response at 3 months after CCRT in locoregionally advanced NPC. Compared with the assessment based on morphological changes, the R2^*^ value can accurately assess the oxygenation status of locoregionally advanced NPC during treatment and can provide a new treatment strategy.

## Data Availability

The datasets generated and analyzed during the current study are not publicly available because they contain information that could compromise the privacy of the research participants, but they will be available from the corresponding author on reasonable request.

## Abbreviations

NPC: nasopharyngeal carcinoma
BOLD: blood oxygen level-dependent
MRI: magnetic resonance imaging
CCRT: concurrent chemoradiotherapy
RG: responding group
NRG: nonresponding group
R2^*^: apparent transverse relaxation rate
R2^*^_preT_: R2^*^ value of tumor before CCRT
R2^*^_post_: R2^*^ value of tumor after CCRT
DWI: diffusion-weighted imaging
DCE: dynamic contrast-enhanced
ICC: intraclass correlation coefficient
AJCC: American Joint Committee on Cancer
TE: echo time
GTVnx: gross tumor volume
GTVnd: cervical metastatic lymph node volume
CTV 1: clinical target volume 1
CTV2: clinical target volume 2
PTV: planning target volume
T1WI: T1-weighted image
TR: repetition time
FOV: field of view
ROI: region of interest
ICC: intraclass correlation coefficient
ROC: receiver operating characteristic curve
AUC: area under the ROC curve
